# Understanding the Course of Critical Illness Through a Lifeworld Approach

**DOI:** 10.1177/10497323211062567

**Published:** 2021-12-27

**Authors:** Stine Irene Flinterud, Asgjerd L. Moi, Eva Gjengedal, Sidsel Ellingsen

**Affiliations:** 1 Faculty of Health Studies, 3459 VID Specialized University, Bergen, Norway; 2 Department of Health and Caring Sciences, 366044 Western Norway University of Applied Sciences, Bergen, Norway and Department of Plastic, Hand and Reconstructive Surgery, National Burn Centre, 60498 Haukeland University Hospital, Bergen, Norway; 3 Department of Global Public Health and Primary Care, 1658 University of Bergen, Bergen, Norway

**Keywords:** aftercare, critical care, follow-up, intensive care, illness trajectory, lifeworld-led care, patient experience, phenomenology, support

## Abstract

An increasing number of individuals receive and survive intensive care treatment; however, several individuals experience problems afterward, which may threaten recovery. Grounded in a lifeworld approach, the aim of this study was to explore and describe what intensive care patients experience as limiting and strengthening throughout their illness trajectories. Ten former intensive care patients were interviewed three to eight months after hospital discharge. Using Giorgi’s phenomenological analysis, a general structure of gaining strength through a caring interaction with others was revealed. The structure consisted of three constituents: feeling safe through a caring presence, being seen and met as a unique person, and being supported to restore capacity. Being met with a humanistic approach and individualized care appeared to be important, and the findings are discussed within the framework of lifeworld-led care. To facilitate improved aftercare of the critically ill, more tailored support throughout the illness trajectory is needed.

## Introduction

Critical illness and intensive care treatment constitute a dramatic threat to a person’s sense of self and security. Each year, approximately 15,000 patients require treatment in Norwegian intensive care units (ICUs). Due to advanced medical treatments, approximately 80% remain alive 30 days after being discharged from the ICU (Buanes et al., 2020). However, a significant number of patients may experience the onset of new physical, mental, or cognitive impairments after intensive care, which is collectively referred to as the post-intensive care syndrome (PICS) (Karnatovskaia et al., 2015; Needham et al., 2012; Rengel et al., 2019). Guidelines and bundles have been established with the aim of minimizing the burden of intensive care through the management of pain, delirium, sedation, sleep, mobilization, family, and patient comfort (Devlin et al., 2018; Marra et al., 2017; Vincent et al., 2016). In addition, ICUs offer interdisciplinary follow-up and rehabilitation, ICU follow-up clinics, and diaries written for patients during their ICU stay, that aim to help patients to fill in their memory gaps from their ICU stay and aid in psychological recovery (Aitken et al., 2013; Svenningsen et al., 2015). In Norway, a survey revealed that approximately 80% of the responding ICUs organized some form of follow-up for intensive care patients, but the offers were not uniform (Moi et al., 2018). The follow-up initiatives also differ between countries, and there is no consensus thus far on the best and most effective aftercare for reducing PICS symptoms (Inoue et al., 2019).

## Background

Individuals who are affected by critical illness continue to live their lives in a multifaceted way and exploring their lifeworld can increase and give depth to their experiences. Patients’ stories from ICU have been widely studied in previous decades, describing memories of chaos, dreams and hallucinations, fear, pain, and other discomforts (Berntzen et al., 2020; Cutler et al., 2013; Egerod et al., 2015; Sanson et al., 2021; Uotinen, 2011). In a synthesis of Nordic research, Egerod et al. (2015) reported how the experience of suffering was evident in intensive care, even though measures had been implemented to improve and make intensive care treatment more humane.

Over the last decades, there has been a shift in focus from survival alone to an increased emphasis on the kind of life patients go back into after surviving a critical illness. Hence, ICU survivorship has been suggested as a concept that encompasses patients’ lives through and after their critical illness (Kean et al., 2017). In this respect, ICU survivorship is more than recovery; it includes moving on and integrating new understanding of one’s own self into the new life after surviving critical illness (Corner et al., 2019; Kean et al., 2017). In addition, each patient’s trajectory has an individual timeframe, which demands individualized care pathways (Kean et al., 2017). Public health care documents recommend individualized and coordinated care pathways, emphasizing the importance of listening to patients’ own voices (National health and Hospital Plan, 2019).

In accordance with this, a humanizing lifeworld approach within health care has been called for, aiming at patients feeling seen and having their needs addressed in depth (Galvin & Todres, 2013). A lifeworld approach is based on the following lifeworld constituents: temporality, spatiality, intersubjectivity, embodiment, mood or emotional attunement, and identity, all of which are mutually dependent and intertwined (Galvin & Todres, 2013, p. 26). Galvin and Todres (2013) propose a framework for a humanizing health care: lifeworld-led care. They highlight that a humanly sensitive health care system needs to take the terms “well-being” and “suffering” into account; they propose an extensive framework in which they outline one lattice for well-being (p. 80) and one for suffering (p. 99). Well-being and suffering are discussed along with the lifeworld constituents and illuminated by dwelling-mobility dimensions (Galvin & Todres, 2013, p. 65). Dwelling is outlined as a peaceful state of being at home in the moment; mobility is described as a sense of future orientation; and the two dimensions are intertwined in dwelling-mobility. However, this study adopts a lifeworld approach; it must be noted that the framework of lifeworld-led care came into consideration after the analysis was completed, and is applied in the discussion chapter.

As studies reveal that former ICU patients may struggle after their ICU stay and experience a lack of individual needs assessment or needs-driven care (King et al., 2019), it is essential to have more research aiming at exploring the trajectories of intensive care patients’ from the onset of critical illness to being back at home, to be better able to develop measures for follow-up adjusted to the patients’ own needs. Therefore, our aim was to explore and describe what intensive care patients experience as limiting and strengthening throughout their illness trajectories to reveal their needs for support and follow-up.

## Method

To gain a deeper understanding of the experiences of intensive care patients, a qualitative design with a phenomenological approach was selected. Giorgi (2009, pp. 89-93) emphasizes the importance of cultivating a phenomenological attitude, which implies being open to the experiences of the participants and ask questions regarding what may appear obvious at first glance. As all the authors are either anesthesia or intensive care nurses and, thus, had some common presumptions, a conscious effort was made to being open to what appeared, taking a step back, and attempting to suspend or put in brackets our pre-understanding in order to see beyond our own immediate understanding (Giorgi, 2009). However, this did not mean that all our pre-understanding could be put in brackets. The authors strived for openness, and, simultaneously, the study was conducted with a caring perspective when asking for the patients’ strengthening and limiting experiences through the trajectory of their illness.

### Participants

The participants were recruited from two ICUs in two university hospitals in Norway. The following inclusion criteria were adopted: age ≥ 18, length of stay ≥ 4 days in the ICU, mechanically ventilated ≥ 48 hours, and being capable of conducting an interview in Norwegian. Ten former patients (four women and six men) consented to participate in the study. The median Simplified Acute Physiology Score II (SAPS II), a score registered after the first 24 hours in the ICU estimating the severity of disease, was 35.5, and they suffered from respiratory failure (*n* = 8), sepsis (*n* = 2), circulatory failure (*n* = 3), trauma (*n* = 2), and gastrointestinal disease (*n* = 2). For patient characteristics, see Table 1. Seven were discharged to go home, while three were discharged to go to other institutions for rehabilitation. They were interviewed three to eight months after hospital discharge.

**Table 1.** Patient Characteristics.

	Age	SAPS II	Days mechanically ventilated	Length of stay in the ICU	Length of stay in hospital
Median	54	35.5	12	14	24
Min–max	26–73	32–55	5–60	7–68	17–138
Interquartile range (IQR)	24	19	18	18	28

### Data Collection

Individual interviews were performed from February 2017 through April 2018. Each interview began with encouragement to discuss the onset of their critical illness, continuing with how they experienced their critical illness trajectory up to the time of interview. The focus was on what they had experienced as limiting and strengthening for their improvement and well-being through their critical illness trajectory to improve their outcome and recovery. An interview guide was used to gain the richest possible data of their entire illness trajectory, from the ICU throughout their hospital admission and onward, including the rehabilitation process and then back at home after discharge. Each interview unfolded based on the stories told by the participants; however, deepening questions on desirable areas were asked (Giorgi, 2009) such as physical, mental, spiritual, activities/work, and social aspects as well as their experience with follow-up services and user involvement to ensure broad descriptions. Overall, the interview guide contained open unstructured questions such as “how did you experience being in the ICU?” or “would you tell me about your everyday life now?” with probing questions such as “will you tell me more about what you experienced as challenging?” or “would you tell me more about what you experienced as important?”. The interviews lasted from one hour to two-and-a-half hours, with the average duration being one-and-a-half hours. The interviews were digitally recorded and transcribed verbatim.

### Data Analysis

The descriptive phenomenological method as described by Giorgi (2009) was used as the analytic tool to capture the structure of meaning of the interviews. The analysis began with reading all transcriptions several times while listening to the recordings to obtain an overall sense of the entire body of material, while simultaneously striving to retain an open-mindedness toward what was being read and heard. After gaining an overall sense of the entirety of the material, the analysis continued by dividing the text from each interview into meaning units using NVivo version 12. The meaning units were then interrogated and transformed into second-order descriptions, containing more direct meanings from a caring perspective (Table 2). In this phase, the analysis moved back and forth from the parts of the material, the meaning units, to the second-order descriptions, in order to identify a structure that best described the material. After identifying the structure of meaning of each participant’s experience, the meanings across all interviews were compared and contrasted, thereby resulting in a general structure of gaining strength through a caring interaction with others, consisting of three constituents: (1) feeling safe through a caring presence, (2) being seen and met as a unique person, and (3) being supported to restore capacity.

**Table 2.** Application of Giorgi’s Method of Analysis from Meaning Units to Constituent.

Meaning units	Second-order descriptions from individual interviews	Second-order descriptions from all interviews	Constituent
It was a guy who had the night shift, and he and I had a nice connection. And I remember laying awake, and I was holding his hand begging him to help me. And he sat with me and, (starts to cry)… Oh, this is really traumatic to think about. But he was, he was such a fabulous guy	Experiencing care and health care professionals that cared for her was good. It was a few people through the trajectory of her illness at the hospital and during rehabilitation that she had a close connection with	The participants described being in an unknown and unsafe existence, where feelings of safety were dependent on health care professionals having a caring attitude	Feeling safe through a caring presence
It was a nurse at night that I don’t…never should have been there. That is the only negative experience. It was the night I got my arterial fibrillation. “Go back to sleep” she said, “you are only nervous” she told me. “Anxiety, this is only your anxiety” she said. What on earth? I have known arterial fibrillation, I can feel it, it had nothing to do with anxiety I told her. Then I asked to see another nurse, and someone else came and checked, and then she contacted the physician who started the treatment	He experienced a situation where he felt unsafe. It was about meeting a health care professional that he felt did not take him seriously. The situation was quickly taken care of by other health care professionals who took over his responsibility		

Furthermore, this general structure was dependent on the three constituents, which encompass the aspect of feeling safe in an unsafe existence, the meaning of self when the body was critically ill, and the specific support required in the process of recovery. We found the constituents to be equally important phenomena that formed the structure of gaining strength through a caring interaction with others. The overall structure is formed by both limiting and strengthening experiences, and the variations are described in the findings chapter.

### Ethical Considerations

Former patients may have had strong and traumatic experiences during their critical illness and hospital stay, and ethical considerations were taken into account in order to prevent anyone from feeling pressurized into participation. A contact person in each ICU identified and sent invitations and information letters to eligible participants. The researchers had no knowledge of the participants until the latter independently made contact and consented to participate in the study. At the beginning of each interview, the interviewer reiterated information regarding the study, emphasizing that the participants had the option to withdraw from the study at any time before it was published. The participants then provided their written consent to participate. In case the participants had questions regarding their ICU stay or they wanted to talk to someone from the ICU regarding issues that surfaced during the interviews, an agreement was made that the appropriate person in the ICU could be contacted after the interview. On one occasion, a participant wanted contact with the ICU, and the interviewer facilitated that contact. The Norwegian Centre for Research Data recommended the study and gave permission to store personal data (ref. no. 397448). Moreover, the data protection officer at each hospital also assessed ethical issues, such as the impact an interview might have on the participants and the routines that must be followed if someone needed support after their interview and gave permission to conduct the study.

### Findings

The analysis revealed a general structure of gaining strength through a caring interaction with others, which was dependent on feeling safe through a caring presence and being seen and met as a unique person while being supported to restore capacity. Waking up to an unfamiliar world and unknown self in the ICU, an unpredictable and insecure lifeworld permeated the participants being, threatening their familiar existence. With fluctuating consciousness, hallucinations, or forgetfulness challenging their lifeworld, the need for a caring presence who saw their individuality formed the foundation for new understandings and different ways of coping. Throughout the illness trajectories, the need for care and support to restore capacity and live on in their new everyday life was crucial.

### Feeling Safe Through a Caring Presence

The impact of different kinds of presence was highlighted through the critical illness trajectory. However, factual presence was not sufficient; strengthening presence that disclosed a caring dimension, had a special quality. A feeling of safety was created by the presence of either their family, health care professionals, or friends. When lacking reference and control over what was happening, the presence of a significant other provided a safe anchor. The adjacency of their close ones connected them to their familiar life, thereby creating a safe space in an unsafe existence:

But as I said, the first part, the first day after I woke up, my husband and daughter were with me. I didn’t want them to go home in the evening, I wanted them to stay. I was convinced that if they left me, I would die.

Furthermore, the presence of health care professionals was also important. However, contrary to the participants’ family, the mere presence of who provided security and comfort, the quality of the presence of the health care professionals was emphasized. The intensive care nurses could be present during fearful moments of deterioration in the ICU, still managing to give the participants a feeling of security despite their serious condition. This could be the feeling of a comforting presence that manifested through touch—that is, by someone holding their hand or massaging their feet. In addition, having ones need for information and knowledge fulfilled appeared to be essential. Understanding what was happening during their critical condition was crucial, and not receiving comforting information was described as challenging and traumatic:

And then they told me: “We have to put you on a ventilator”. And I remember thinking that I was going to die. That was the feeling I got. And I don’t know if they didn’t dare to tell me, or maybe they didn’t know, I don’t know, but I would have needed someone to tell me: “This is going to be all right, you are in good hands, this is for your own good”, but I cannot remember receiving any kind of comforting information.

Both within the hospital and the subsequent rehabilitation, participants expressed the significance of a certain health care professionals—the health care professionals that went the extra mile, doing something beyond what was expected, was what made a huge difference to the participants. The presence of such professionals was comforting and provided them with security despite their challenges when recovering:

It is strange, but when I look back on the ICU stay, I become happy. Because there were some fantastic people there, and of course I felt afraid, but still very safe. And, I had a couple of nurses by my side for the most dramatic period, and you get to know them. And I felt they did so much more for me than they had to.

As time went on and the recovery proceeded, the quality and form of the presence they needed to feel safe changed. After being discharged to go home, the participants’ family took over as the main caregivers, helping and supporting them. To make everyday life work for themselves, they put their safety in the hands of their family. Being discharged to go home was described with ambiguous emotions—both longing to be home in their everyday life but simultaneously acknowledging that they were not the same as they were earlier:

I had mixed feelings about coming home. Sort of like: How on Earth will I manage? At the same time, it was really nice to come home, so something in between fear and joy.

Furthermore, when recovering, their social network became increasingly important. Feeling love and care from friends that visited or came over with food was greatly appreciated. However, when balancing between being dependent and independent, the helping and supporting role of their family and friends could occasionally be challenging and made the participants long for their everyday life to be as it was before the illness.

In summary, feeling safe through a caring presence fathoms a variety of presence that gave the participants a feeling of safety through the trajectory from waking up critically ill in the ICU and onward throughout hospitalization and eventually to being back at home. The caring quality of the presence was important, individualized to the participants’ needs, providing safety and comfort through information and support.

### Being Seen and Met as a Unique Person

Throughout their illness trajectories, feelings of their own vulnerability were expressed by the participants through stories of how much they valued being seen and met as unique individuals. To be dependent on the health care professionals had put the participants in a vulnerable situation. Losing control over themselves could be difficult, but being met with respect made it easier:

I don’t like being dependent on others, I find it difficult asking for help. But in the ICU, you are totally dependent on others, and you are treated with so much respect that you just have to let go and think: “Ok, it’s ok. Even for someone like me, who likes to do things myself.”

However, not feeling respected and not being taken seriously, could cause anger and bring up upsetting emotions. It could be events such as calling for the nurse at night, telling about a new onset of arrhythmia, and being told to relax because the nurse said it was only caused by anxiety, or not being taken seriously or feeling respected during their treatment:

The first part was very traumatic for me. I had to use this ventilator mask, covering my whole face. Then they asked me all these questions that I was unable to answer or disagree with if I didn’t want whatever it was they were offering. It was as if I did not have my own free will, well, I did, but I could not express it. In addition, the mask felt like coercion. I understood that I had to wear the mask for a set amount of time, but I did not feel they complied with the timeframe. And again, in my profession, being aware of time is extremely important. Therefore, I have some knowledge about how long 20 minutes is. And in my opinion, they did not comply, and I was really upset.

Being seen and met as individuals with unique personalities and traits was highlighted. Discussing everyday matters, their faith, or making jokes with the health care professionals looking after them was valued and viewed as essential when going through a difficult time. Humor was described as a coping mechanism when experiencing something challenging and also as a means of revealing their personality and connecting with the health care professionals looking after them:

I thought it was very nice with the men (male nurses). My family and I, we like to joke a lot. And with the men, they sort of became more like my friends, we could joke around even though it was a serious situation. A bit of morbid humor, which was fun.

However, their experiences of being seen and met as unique individuals could be challenged when they themselves were not the same as they used to be. Their critical illness, sedation, or medical treatments could make them act different from how they were before the illness, and it was challenging for them to experience losing control over themselves and their appearance. Being told in retrospect that they had behaved badly, such as hitting or attempting to fight against the health care professionals who were trying to help them, felt disturbing. Moreover, experiencing amnesia was also described, an experience that could be challenging for a long time after discharge:

I don’t remember anything from the first couple of weeks in the hospital; they are completely gone. I don’t remember a thing, and it really bugs me. I was looking at my phone just now, and I discovered that I had sent some messages during that time, but I cannot remember a thing about it. It is completely gone, and I think it is awful, it is really bugging me.

Having been through critical illness, the impact of receiving attention from others was described. Getting attention and feeling seen and met by significant others were highlighted as important and made the participants feel good. However, this stood in contrast to the attention from people who were more peripheral. Getting more unwanted attention after going home, such as feeling like others are staring at them or initiating conversation because of what had happened was mentioned as a challenging experience. To minimize the risk of inadvertently meeting peripheral acquaintances, the participants described implementing measures such as going shopping in a different town or booking the first appointment of the day when going to the doctor.

In summary, being seen and met as a unique person included the dimensions in which their personalities and sense of selves were affected. Their dependence on the health care professionals made them feel vulnerable and reliant on caretakers who saw them as individuals with unique personalities. Being treated with respect, being able to joke around, and feeling genuine concern from others supported their own recognition of self.

### Being Supported to Restore Capacity

When waking up being critically ill, a fight for getting back to one’s familiar life began. Finding inner strength and motivation and obtaining external support were both mentioned as important factors on the path to recovery. Physical weakness and mental challenges due to their critical illness affected them greatly. In the hospital, it could be experiences such as being locked inside their own body, unable to communicate or move, or not being able to do what they wanted without help, such as peeling an orange or getting out of bed on their own:

The first days, what I noticed was the clarity in my head. But then, my body would not function. It would not cooperate, which made me feel almost like being imprisoned in my own body.

These experiences motivated the desire in them to recover and begin their training and rehabilitation; it was in such a situation that the personal history and life experience assumed new importance. Personality traits such as being positive, stubborn, or patient were described as imperative—for example, finding strength to go out for a walk every day despite the rain and snow in order to get better or creating their own exercise program when the rehabilitation offered by the health services were suboptimal.

Furthermore, after discharge from hospital, a few were transferred directly to a rehabilitation center, and others were offered different courses or exercise with, for example, physiotherapists at home, while a few were discharged without such offers. Regardless of whether they had received an offer, they sought more tailored knowledge on the activities of daily living and individually adjusted offers:

No, there hasn’t been any offer or someone asking if we needed help. And in that context, it is important to state that neither me nor my wife were capable of asking for help at that time. So, someone has to come and offer it; you are not able to do it yourself, you are just not able.

Thus, physiotherapists and other health care professionals provided security and motivation—for example, informing the participants how far they could push themselves during workouts or informing them of the possible side effects of their new medication. However, such offers did not always fit their needs; moreover, when they received an offer that did not suit them, the system was stringent with little flexibility:

I was in too much pain; my breast had not recovered. There should have been flexibility with the rehabilitation, if I could have waited a couple of weeks before I started, so the pain in my chest had decreased. Then I would have benefited more from the rehab. But that flexibility did not exist.

In addition to wanting physical recovery, the participants emphasized the importance of recovering mentally. Traumatic experiences had left their mark, and they required help in handling their experiences at an emotional level. The participants expressed the need to discuss their experiences after the most critical phase was over, whether it was talking to their family, friends, or health care professionals. However, talking to family or friends was helpful only up to a certain point, but the possibility of discussing their dramatic experiences with health care professionals during or after hospitalization was described as a possible means of helping them handle their experiences and helping with the mental challenges in the aftermath:

If I could have wished for something during the hospital stay, it might be a psychologist. Maybe he could help me, I don’t know, maybe he would understand what I was talking about. (…) Then maybe I could have avoided becoming so afraid of sleeping, and with the claustrophobia... I do know it is nothing to be afraid of, but when I felt I was having problems breathing, I started hyperventilating. And then my anxiety multiplied every time.

Furthermore, the diary that participants had obtained from the ICU was described with a range of emotions. While some were touched and had tears in their eyes while discussing it and described using it a lot in their subsequent coping, others had not read it for months after discharge, saying that it did not feel right. Thus, while the diary could be of tremendous help, it could also evoke difficult and disturbing feelings, which made it challenging to read:

Then the diary came, and I’m thinking, that diary should not be handed out without a conversation with someone. Because the questions I had, they were only amplified. The diary does not give me any explanation. Looking at yourself from outside, lifeless, and your family around the bed. I don’t know… All of the uncertainty and fear, all of these bad feelings, they surfaced with those pictures.

After the critical illness, a new perspective on life had emerged, and to recover meant working toward becoming the best version of themselves they could be. Descriptions of a desire to return to normal everyday life stood out even though this everyday life was not always the same as before their critical illness. They wanted an everyday life they felt comfortable with in their new situation, implementing the newfound perspectives in their ongoing lives even though this could be challenging:

But then suddenly your everyday life hits you. I was thinking that I should live a bit different and stop working so much. But then the bills come, and you must work to be able to continue living like you did. It is difficult, because I sort of want to live a bit different, but I also want to keep what I got. But the worst thing I could do is to continue living exactly like I used to.

When taking back their everyday life, being able to contribute, being useful and going back to work were described as meaningful. Employers who customized the work of the participants according to their situation and were flexible regarding their jobs were highly appreciated.

Briefly summarized, being supported to restore capacity was important on the path to recovery. They experienced challenges and required individually tailored help and support throughout their recovery trajectory. However, recovering did not mean getting back to how they had been before but rather regaining a familiar foothold and becoming the best version of themselves in their new everyday life.

## Discussion

The participants described how gaining strength through a caring interaction with others was crucial throughout their illness trajectory. Feeling safe through a caring presence, being seen and met as a unique person, and being supported to restore capacity were all essential constituents that formed the general structure. Critical illness may be experienced as a biographical disruption; it is an unwelcomed breach in a person’s history, where holistic care that considers the personhood, situation and life purposes of each patient is required (Tembo, 2017). Our argument is that a humanistic view of the needs of the patients involved in critical care and follow-up appears to be necessary, and our findings are discussed with the framework of lifeworld-led care. Short examples from the dimensions discussed are presented in Table 3.

**Table 3.** Experiential Domains of Well-being and Suffering.

	Dwelling	Mobility	Dwelling-Mobility
Spatial dimension of well-being	Feeling a*t-homeness* through the presence of a caring other		
Spatial dimension of suffering	Being in *exile* when waking up in the ICU feeling unhomelike and not belonging	Being in a *spatial imprisonment* when waking up in the ICU unable to move	
Intersubjective dimension of well-being	Feeling a sense of *kinship and belonging* in interaction with some of the health care professionals		
Intersubjective dimension of suffering	Feeling *alienated isolation* when experiencing fear and loneliness		
Temporal dimension of well-being	Through the presence of their family or health care professionals, the participants experience a *present centeredness* thereby improving their well-being	The participants experience a *future orientation* wanting to recover to a familiar everyday life	Experiencing *renewal* involves how the participants incorporated their experiences in their ongoing life
Temporal dimension of suffering	Being in an *elusive present* when waking up feeling an unsettledness		
Identity dimension of well-being	The *I am* describe how the participants valued being met in regards to who they are	The *I can* describe how the participants found strength within themselves to continue rehabilitation	
Identity dimension of suffering		The *I am unable* describes how some participants were unable to act on own needs and would have needed someone to help them	

The words in italics are terms from the lifeworld-led care framework (Galvin & Todres, 2013).

Svenaeus (2000, p. 100) describes health as “homelikeness” and illness as “unhomelikeness.” When healthy, we are “homelike” in the world with an attuned understanding (Svenaeus, 2000); when one is attuned, one has access to a familiar world with known meaning structures (Svenaeus, 2011). However, in illness, one may be “out of tune,” where our being-in-the-world feels unhomelike (Svenaeus, 2000, p. 97). Describing the unhomelike attunement, Svenaeus (2000) draws on Heidegger’s notion of uncanniness, which Heidegger (1962, p. 233) described in relation to the experience of not-being-at-home when everyday familiarity might collapse in the face of anxiety. When waking up in the ICU, the participants described being in an unfamiliar world, finding themselves in a new and unhomelike situation while being critically ill. Thus, it can be said that their *spatial dimension* was affected. Moreover, what Svenaeus (2000) calls “unhomelikeness” may be what Galvin and Todres (2013) illuminates as “exiled” within the spatial dimension of dwelling suffering. It describes how one may feel in an alien place or not belonging, which may describe the participants’ experiences when waking up in the ICU, in an unknown environment, having to surrender to and be dependent on technical equipment and health care professionals for survival, and onward when working their way back through recovery. Simultaneously, when waking up in the ICU, descriptions of how their bodies did not obey or function like they were used to were common, which may indicate the experience of being in a spatial “imprisonment” within the spatial dimension of mobility suffering (Galvin & Todres, 2013, pp. 99-100). Bentsen et al. (2020) found that a gap existed between the patients’ comfort needs and the nursing care provided. Moreover, in a study investigating interhospital transfers of ICU patients, Karlsson et al. (2019) discuss how the patients may recede into the background and become “invisible” to the health care professionals as technology and technical equipment dominate. This may be examples of how the spatial dimension of suffering is evident for ICU patients; however, these are not constant dimensions, and, in our study, we found examples of going from suffering to well-being in the spatial dimension—what is called “at-homeness.” This could be the presence of a caring other holding one’s hand in the ICU or being given information regarding physical workout in their rehabilitation to increase the sense of security, which could take the participants from suffering to well-being and a situation of “at-homeness” (Galvin & Todres, 2013, p. 82).

The term well-being has recently been explored in a review that highlights the barriers and facilitators to well-being in an ICU setting (Halvorsen et al., 2021). Well-being was found to be a multidimensional experience, in which barriers were linked to physical and emotional stressors, the ICU environment and insecurity; the facilitators of well-being included a caring and relational environment (Halvorsen et al., 2021). It appears that the spatial dimension and the environment surrounding the patients in the ICU and onward throughout the recovery phase affects the patients on the path between well-being and suffering; it is important to consider this when working toward improving health care practice for intensive care patients.

The *intersubjective dimension* was strongly highlighted in our findings. When the participants’ life was at stake, and an unhomelike situation permeated their whole existence, the intersubjective dimension became evident. The health care professionals connected and performed caring acts that often went beyond what the participants expected. The *intersubjective dwelling in well-being* is described a “sense of familiar interpersonal connection” (Galvin & Todres, 2013, p. 86), where a sense of kinship and belonging is emphasized, as described by the participants. Caring acts brought the participants closer to a feeling of “homelikeness” within their state of uncertainty and “unhomelikeness.” When interacting with patients, it is the health care professionals’ responsibility to understand and help patients on their way from an unhomelike reality and bring the patient closer to a homelike actuality (Svenaeus, 2011). A caring presence was significant for our participants’ well-being as they recovered. Hawley and Jensen’s (2007) study of what “making a difference” means to critical care nurses describe how acting in a way that supports the patients, so their unbearable and inhumane situation becomes bearable and humane, was highlighted. Small gestures, such as a warm greeting or touch could be one way of making a difference and the inhumane situation humane for the critically ill patients (Hawley & Jensen, 2007).

However, our findings also show how a few participants felt isolated and alone, and would have needed a caring other by their side—an aspect that Galvin and Todres (2013, pp. 104-105) call “alienated isolation.” In a recent study by Sanson et al. (2021), a therapeutic relationship with health care professionals was the most important factor influencing whether patients carried back good or bad memories from the ICU. We argue that if health care professionals are interested in the individual, seeking information about who they are, and encourage their families to bring the patients’ personal belongings to the hospital, it may facilitate the creation of a common intersubjective space between the patient and the health care professional which may bring the patient toward a more homelike actuality.

The *temporal dimension* was also found to be affected. When ill, the present time becomes accentuated (Svenaeus, 2011), which was highlighted by the participants when describing that their temporal dimension shrunk to the present time when being critically ill, and aspects like the presence of family became important. As part of the temporal dwelling-mobility of well-being, Galvin and Todres (2013, pp. 83-85) describe mobility as “future orientation”, and dwelling as “present centeredness.” Simultaneously, temporal dwelling within suffering is described as the “elusive present” (Galvin & Todres, 2013, p. 102). When waking up, the participants described feeling like they were in limbo, uncertain and unsafe. Within the “elusive present,” an unsettledness and temporal suffering is present, which may be an example of the participants’ descriptions when waking up in the ICU. The participants longed for a caring other, and descriptions of how important this presence was could be seen as a means of moving from an “elusive present” to “present-centeredness,” where one is absorbed in the here and now. Within the “present-centeredness,” the presence of a caring other was their anchor and lifeline, regardless of whether it was their family or a caring health care professional. This is also highlighted by Berntzen et al. (2020), who described how presence of family seemed to anchor the patients to reality when everything else was experienced as chaotic. In our study, the highlighting of fear and anxiety when not feeling the presence of a caring other shows how moving from well-being to suffering can be rapid and may be like balancing on a fine line.

Nevertheless, as their recovery proceeded, they moved from dwelling toward mobility with a future orientation, ending with the temporal dwelling-mobility in well-being called “renewal” (Galvin & Todres, 2013, pp. 84-85). “Renewal” incorporates how the participants described embracing their experiences, taking the experience with them, and integrating it with the desire of leading a different and evolved life after their critical illness. Surviving intensive care treatment is more than just recovering, and the critical illness may challenge one’s identity, bringing about a “new normal” afterward (Kean et al., 2017). Critical illness survivors have described how they had to integrate their new and old selves (Egerod et al., 2020). In a grounded theory study of patients with PICS symptoms, “embracing the new vulnerable self” emerged as a core category (Kang & Jeong, 2018), thereby revealing how patients with PICS symptoms experience recovery as a continuum in which a new normality emerges after the critical illness. These findings are supported by ours, where the participants described how they desired to integrate their experiences into their new everyday life, subsequently becoming a new version of themselves on the other side, which is in line with the proposed term ICU survivorship and “renewal” (Galvin & Todres, 2013; Kean et al., 2017). Hence, support in redefining and becoming homelike within their new life after critical illness may be a step in the right direction in aftercare and follow-up of patients who have needed intensive care treatment.

Furthermore, the *identity dimension* was illuminated through the importance of being seen and met as a unique person. The participants valued being seen and met as themselves even though their appearance may be different from what it was before the critical illness. The significance of being able to reveal their personalities, like through using humor, made them feel seen and met as unique individuals. In accordance with our findings, to be valued as a unique individual, and being seen, believed in, and listened to is also highlighted in a review of Nordic research on dignity in health care settings (Lindwall & Lohne, 2020). From a health professional’s viewpoint, this may be illuminated by what Todres et al. (2014) calls “caring for insiderness,” which illustrates how health care professionals must strive for understanding the insider perspective of the patients. However, they underscore how this does not mean that they must know everything about the patients; rather, they need to possess an open attitude and be interested in the patients’ lifeworld (Todres et al., 2014), which is described in our findings of how the participants valued the health care professionals’ interest in them as unique persons. This is supported by Tate et al. (2012), who found that knowing the patient and their individual preferences was important when responding to the patient’s anxiety. In addition, Hawley and Jensen (2007, p. 666) also highlight the importance of acknowledging the humane-ness by seeing “the person inside the body”.

Within the *identity dimension of well-being*, identity mobility is called “I can,” and identity dwelling is called “I am” (Galvin & Todres, 2013, pp. 90-93). “I am” is described as where our general sense of self lies, our fundamental sense of being, without other ways of describing ourselves as being someone or something (Galvin & Todres, 2013). In our study, being valued and met with regard to identity was of tremendous importance and was described as supportive and constructive for participants’ motivation and coping within their difficult situation. The “I can” describes how one feels when on their path toward something that one values or wants or is capable of doing (Galvin & Todres, 2013, pp. 90-91). When waking up critically ill, a fight for getting back to a more familiar life begins, and the participants in our study received a variety of rehabilitation offers. While some offers met their needs, several did not, and their inner strength and own capacities became important when they themselves had to adjust and push on to either get the rehabilitation they needed or do it themselves, hence, being in the “I can” where they found strength within themselves. However, others highlighted how they were not able to do this, which might be what is called “I am unable” in the identity mobility of suffering. To ensure that patients who find themselves being “unable” get adequate help, the health care must be tailored to individual rehabilitation that meets the patient’s needs. This is also a goal within public health care documents (National health and Hospital Plan, 2019) and is supported by previous research that highlight how survivors of critical illness may experience barriers post ICU discharge and have difficulty coordinating the follow-up they need (Calkins et al., 2021; Maley et al., 2016).

### Strengths and Limitations

The interviews conducted in this study encompass the entire illness trajectory, from the incident causing the ICU stay and throughout the recovery and discharge to home, which provided an in-depth understanding of the participants’ experiences. The findings provide insight regarding similar populations, but simultaneously need to be considered and interpreted within the context of Norwegian public support systems. The participants’ length of stay in the hospital, trajectories of recovery, and time period from discharge to interview differed among participants. However, the median SAPS score (35.5) is comparable to the score of intensive care patients in Norway, being in the mid-30s in 2019 (Buanes et al., 2020). Thus, the severity of illness in our participants appears to be comparable to the general Norwegian ICU population. On the other hand, the variations in the trajectories of illness may strengthen the results when identifying a general structure of meaning across different contexts. In order to improve rapport and create a safe environment during the interviews, the participants were able to choose where they wanted the interviews to take place. All but one participant wanted the interview in their home. The interviews contained rich descriptions, and the participants appeared relaxed and willing to share their experience, which may have been facilitated by being at home in a familiar environment. Individual interviews were conducted with both former patients and their next of kin, and this might also have influenced the participants’ stories. However, the interviewer highlighted that all participants should talk about their own story from their own perspective, and we found that they were open and honest in the interviews, without focusing on the experiences of their next of kin.

## Conclusion and Relevance to Clinical Practice

The importance of gaining strength through a caring interaction with others was an essential finding in this study and included the following constituents: feeling safe through a caring presence, being seen and met as a unique person and being supported to restore capacity. Waking up in an unhomelike situation in the ICU, the participants highlighted how a caring relational presence helped them to recapture a more homelike situation and move from suffering to well-being. To receive individualized attention, a humanistic approach to care and support was crucial on their path to recovery. Humanistic theories such as lifeworld-led care might enable health care professionals to reflect on the disruptions of life history of patients caused by critical illness and how to support patients’ recovery back into a meaningful life. Even though life-saving treatments are crucial in the ICU, a humanistic and caring attitude of supporting patients’ development of inner strength, potential capacity, and the integration of their experiences and aftereffects from the ICU into their ongoing lives appears essential.

There is a need for a health care and follow-up offer that takes each individual patient’s needs into account to help facilitate an individualized follow-up trajectory. As the rehabilitation offered may not fit a patient’s needs and they may be dependent on their own resources when working their way back to a meaningful life, it might be appropriate to have a coordinated service that follows the patients throughout their trajectories both within the hospital and further on when discharged to go home. Then, a coordinator might be able to ensure that the offers fit the patients’ needs and might also facilitate the identification of those who do not themselves have the strength, resources, or family to help them with their needs for rehabilitation and support when the public offer is missed or is entirely absent.
